# ﻿A taxonomic study of *Cheiloneurus* Westwood (Hymenoptera, Encyrtidae) from China

**DOI:** 10.3897/zookeys.1198.118944

**Published:** 2024-04-24

**Authors:** Haiyang Wang, Wenyu Cui, Chunxiang Xi, Xinyu Cao, Weiqiong Li, Guohao Zu

**Affiliations:** 1 College of Horticulture and Landscape, Tianjin Agricultural University, Tianjin, 300392, China Tianjin Agricultural University Tianjin China

**Keywords:** Chalcidoidea, Cheiloneurini, new species, parasitoids

## Abstract

Fourteen species of *Cheiloneurus* from China are studied. *Cheiloneurusguangxiensis* Zu, **sp. nov.**, is described as new to science, and *C.boldyrevi* Trjapitzin & Agekyan, 1978, *C.bouceki* Anis & Hayat, 2002, *C.gonatopodis* Perkins, 1906, and *C.hadrodorys* Anis & Hayat, 2002 are newly recorded from China. A key to Chinese species based on females is also presented.

## ﻿Introduction

The genus *Cheiloneurus*, established by Westwood (1833), with *C.elegans* Dalman as it type species, is very large and diverse (Noyes and Hayat 1984). It encompasses 151 recognized species worldwide (Noyes 2019). All *Cheiloneurus* species exhibit hyperparasitic behavior, targeting a broad spectrum of parasitoid wasps (Trjapitzin and Zuparko 2004). In China, nine *Cheiloneurus* species have been documented, primarily parasitizing Aphelinidae, Encyrtidae, and Dryinidae, engaging in hyperparasitism on various insects, including Hemiptera (e.g. Coccidae, Pseudococcidae) and Diptera (e.g. Drosophilidae) (Xu and Huang 2004; Li et al. 2020; Wang et al. 2023). *Cheiloneurus* is characterized by distinctive features, including the arrangement of setae in the basal cell of the fore wing, typically infuscate fore wings, the presence of an apical tuft of setae on the scutellum, and a hypopygium that does not reach the apex of the gaster (Noyes and Hayat 1984).

Various taxonomists, including Girault (1915) in Australia, Trjapitzin (1989) in the Palaearctic, Hayat and Veenakumari (2017) and Anis and Hayat (2002) in India, Trjapitzin and Triapitsyn (2008) in the New World, and Xu and He (2003) and Shi et al. (1994) in China, have contributed significantly to the taxonomy of the genus *Cheiloneurus*. In this study, we present a comprehensive taxonomic examination of nine known species, introduce one new species, and newly report four other *Cheiloneurus* species from China. The primary objective is to enhance the precision of identifying Chinese *Cheiloneurus* parasitoids. Additionally, we furnish a key for the female species of *Cheiloneurus* in China.

## ﻿Materials and methods

Photographs of specimens in ethanol were taken using a Canon EOS 80D camera equipped with a Laowa 25 mm lens. A Motic SMZ-168 stereomicroscope was used to dissect specimens, which were mounted on slides according to Zhang et al. (2022) and Noyes (1982). Slide-mounted specimens were photographed with a digital camera attached to an Olympus BX51 running Olympus cellSens Standard v. 1.18. The pictures were synthesized through Helicon Focus v. 6 and processed using Photoshop 2020. Each characteristic part was measured using an Olympus CX21 equipped with a micrometer in the eyepiece. All materials were deposited in the insect collections of Tianjin Agricultural University (**TJAU**), China.

Morphological terminology and abbreviations were based on Noyes (2010). The subsequent list provides the employed abbreviations:

**AOL** minimum distance between a lateral ocellus and median ocellus

**F1–6** funicle segments 1–6

**FV** minimum frontovertex width

**FWL** fore wing length

**HWL** hind wing length

**HWW** hind wing width

**MS** malar space

**MT** mid tibia

**OCL** minimum distance between a lateral ocellus and occipital margin

**OD** longest diameter of an ocellus

**OL** ovipositor length

**OOL** minimum distance between a lateral ocellus and the corresponding eye margin

**POL** minimum distance between lateral ocelli

**SMV** submarginal vein

**MV** marginal vein

**PMV** postmarginal vein

**SV** stigmal vein

**BMNH**The Natural History Museum, London UK

**HAUZ**Department of Plant Protection, Henan Agricultural University, Zhengzhou, China

**LUNZ**Department of Entomology, Lincoln University, Canterbury, New Zealand

**USNM**United States National Museum of Natural History, Washington DC, USA

**BPBM**Bernice Pauahi Bishop Museum, Honolulu, Hawaii

**ZAUC**Institute of Applied Entomology, Zhejiang University, Hangzhou, Zhejiang, China

**ZAFU** Department of Plant Protection, School of Agriculture and Food Science, Zhejiang Agriculture & Forestry University, Huangzhou, Zhejiang, China

**ZDANU** Department of Zoology, Aligarh Muslim Uiversity, Aligarh, India

**NIES**National Institute of Agro-Environmental Sciences, Ibaraki, Japan

## ﻿Results

### ﻿Key to Chinese species of *Cheiloneurus* (females)

**Table:** 

1	Scutellum without a tuft of bristles at apex	**2**
–	Scutellum with a tuft of bristles at apex	**4**
2	Fore wing hyaline	***C.lateocaudatus* (Xu & He, 2003)**
–	Fore wing infuscate	**3**
3	F4 yellowish white; linea calva closed posteriorly by several lines of setae	***C.hadrodorys* Anis & Hayat, 2002**
–	F4 dark brown; linea calva open posteriorly	***C.exitiosus* Perkins, 1906**
4	Scape at least 4.75× as long as wide	**5**
–	Scape not more than 4.5× as long as wide	**8**
5	Fore wing hyaline towards base, at apex and along anterior margin distad of venation	**6**
–	Fore wing with basal cell almost completely hyaline, small area at apex of venation and area on opposite margin hyaline	**7**
6	F5–F6 yellowish white; ovipositor not more than 1× as long as mid tibia	***C.gonatopodis* Perkins, 1906**
–	F5–F6 dark brown; ovipositor at least 1.20× as long as mid tibia	***C.bouceki* Anis & Hayat, 2002**
7	Clava not more than 1.78× as long as wide, slightly shorter than F4–F6 combined; head not more than 0.80× as wide as long	***C.nankingensis* Li & Xu, 2020**
–	Clava at least 2.72× as long as wide, slightly longer than F3–F6 combined; head at least 0.90× as wide as long	***C.elegans* (Dalman, 1820)**
8	F6 black	**9**
–	F6 entirely white or mixed with brown	**11**
9	Fore wing hyaline towards base, at apex and along anterior margin distad of venation; frontovertex not more than 1/10 width of head	***C.axillaris* Hayat, Alam & Agarwal, 1975**
–	Fore wing with basal cell almost completely hyaline, small area at apex of venation and area on opposite margin hyaline; frontovertex at least 1/9 width of head	**10**
10	F4–F5 entirely whitish	***C.claviger* Thomson, 1876**
–	F4–F5 whitish with a brown stripe on ventral margin	***C.chinensis* Shi, 1993**
11	F1 entirely whitish	**12**
–	F1 yellowish-brown	**13**
12	Clava as long as F4–F6 combined; pedicel as long as F1	***C.quercus* Mayr, 1876**
–	Clava as long as F3–F6 combined; pedicel at least 1.54× as long as F1	***C.sinensis* Özdikmen, 2011**
13	Scape at least 3× as long as wide; fore wing at least 3× as long as wide	***C.boldyrevi* Trjapitzin & Agekyan, 1978**
–	Scape not more than 2.44× as long as wide; fore wing not more than 2.73× as long as wide	***C.guangxiensis* Zu, sp. nov.**

#### ﻿ Cheiloneurus axillaris

Taxon classificationAnimaliaHymenopteraEncyrtidae

Hayat, Alam & Agarwal, 1975

299C3F36-CF2A-545C-8381-E55EBC44A2B6

Figs 1–3

Cheiloneurus axillaris Hayat et al. 1975: 47. Holotype ♀, BMNH, India, Maharashtra, not examined.
Cheiloneurus axillaris Anis and Hayat 2002: 171–172.
Cheiloneurus axillaris Xu and He 2003: 103–104, examined plates.

##### Material examined.

China – Yunnan • 2♀; Longchuan; 24°10'59"N, 97°47'32"E; 1336 m elev.; 27 Ari. 2013; Guo-Hao Zu, Xiang-Xiang Jin, Chao Zhang leg.; by yellow pan trapping; TJAU-YN-CHE-001 to 002.

**Figures 1–9.**
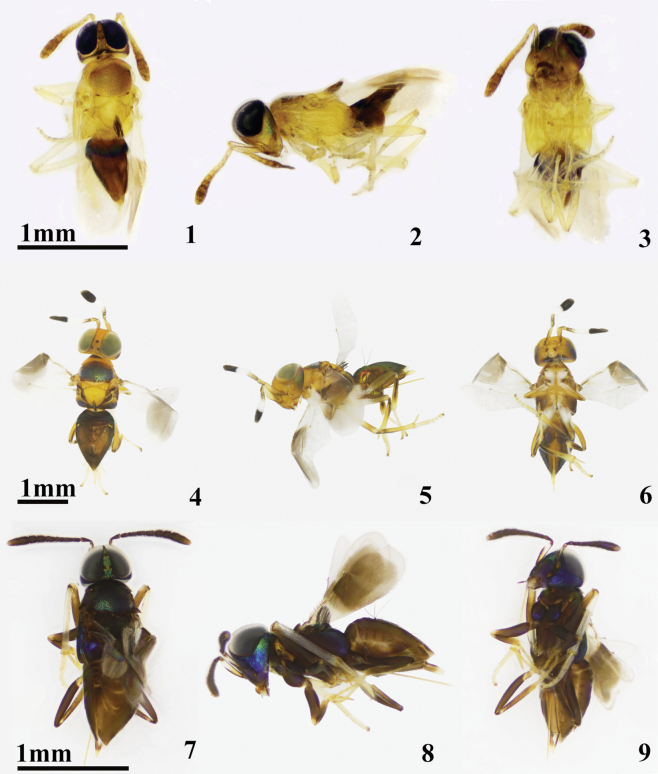
**1–3***Cheiloneurusaxillaris* ♀ **1** dorsal habitus **2** lateral habitus **3** ventral habitus **4–6***Cheiloneurusboldyrevi* ♀ **4** dorsal habitus **5** lateral habitus **6** ventral habitus **7–9***Cheiloneurusbouceki* ♀ **7** dorsal habitus **8** lateral habitus **9** ventral habitus.

##### Diagnosis.

**Female**. Length, excluding ovipositor, 1.78–1.89 mm; head dark brown, gena with bluish-green metallic luster; antennal scape brown, with an apical white area; pedicel yellow; funicle yellowish brown; clava dark brown. Legs pale yellow to white.

##### Description.

See Hayat et al. (1975).

##### Host.

Coccidae: *Ceroplastesjaponicus*, *Pulvinariapsidii*; Margarodidae: *Icerya* sp. (Hayat et al. 1975).

##### Distribution.

China (Fujian, Yunnan), Bangladesh, India.

#### ﻿ Cheiloneurus boldyrevi

Taxon classificationAnimaliaHymenopteraEncyrtidae

Trjapitzin & Agekyan, 1978

DF93B780-1855-5387-A853-22CFA263659F

Figs 4–6 , 10–16

Cheiloneurus boldyrevi Trjapitzin and Agekyan, in Trjapitzin 1978: 309–310. Holotype ♀, ZISP, Russia, not examined.
Cheiloneurus boldyrevi Guerrieri and Viggiani 2005: 312–313.
Cheiloneurus boldyrevi Japoshvili et al. 2016: 367.

##### Material examined.

China – Tianjin • 1♀; Xiqing, Tianjin Agricultural University; 39°5'21"N, 117°5'38"E; 13 m elev.; 12–30 Jun. 2023; Hai-Yang Wang, Xin-Yu Cao leg.; by Malaise trapping; TJAU-TJ-CHE-001.

##### Description.

**Figures 10–16.**
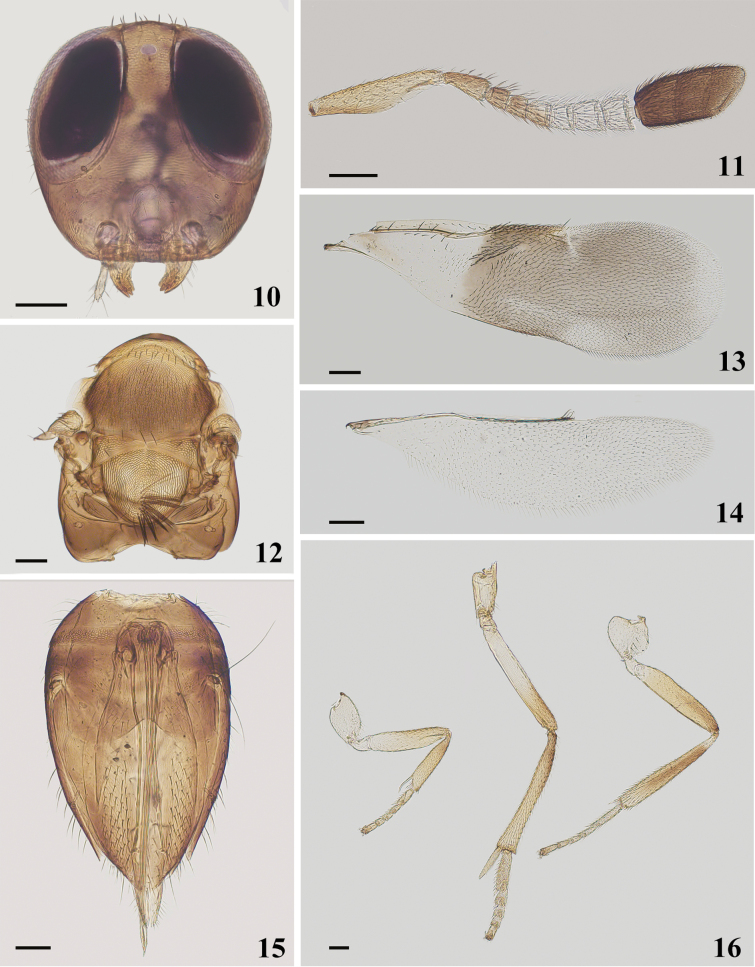
*Cheiloneurusboldyrevi* ♀ **10** head **11** antenna **12** mesosoma **13** fore wing **14** hind wing **15** metasoma **16** legs. Scale bars: 100 μm.

Length, excluding ovipositor, 2.88 mm. Head generally brown; gena with metallic-green luster; antennal scape orange, pedicel and F1–F2 brown, F3 brown with whitish spot in the upper corner of the fore margin, F4–F6 white, clava black, apex paler; basal half of pronotum dark brown and apical half orange, mesoscutum dark brown with metallic-green luster, axilla and scutellum orange, propodeum black, legs orange, fore coxa and basal half of femur, mid basal half of femur, hind coxa, base and apex of tibia whitish. Frontovertex 0.19× head width; eye height 2.06× malar space (Fig. 10); antennal scape 4.14× as long as wide; pedicel 2.22× as long as wide and longer than F1, funicle 6-segmented, F1–F3 and F5 longer than width, F4 subquadrate, F6 wider than long; clava 3-segmented, 2.26× as long as width, longer than F4–F6 combined (Fig. 11); fore wing 3.33× as long as wide; linea calva not interrupted and open posteriorly (Fig. 13); ovipositor (Fig. 15) 1.36× as long as mid tibia (Fig. 16), distinctly exserted.

##### Host.

Dryinidae: *Neodryinustyphlocybae*; Syrphidae: *Paragus* sp., Syrphidae sp.; Flatidae: *Metcalfapruinose* (Guerrieri and Viggiani 2005).

##### Distribution.

China (Tianjin), Armenia, Bulgaria, France, Georgia, Greece, Iran, Italy, Moldova, Netherlands, Portugal, Russia, Spain, Tadzhikistan, Ukraine, Uzbekistan.

##### Comments.

This is the first record from China.

#### ﻿ Cheiloneurus bouceki

Taxon classificationAnimaliaHymenopteraEncyrtidae

Anis & Hayat, 2002

EECFD0FD-CC80-55DA-B90A-D2525DE78C1C

Figs 7–9 , 17–22

Cheiloneurus bouceki Anis and Hayat 2002: 164–165. Holotype ♀, BMNH, India-Karnataka, not examined.

##### Material examined.

China – Guangxi • 26♀; Qinzhou, Beibu Culf University; 21°53'53"N, 108°36'56"E; 24 m elev.; 06–13 Oct. 2019; Wen-Quan Zhen leg.; by Malaise trapping; TJAU-GX-CHE-001 to 026.

##### Description.

**Figures 17–22.**
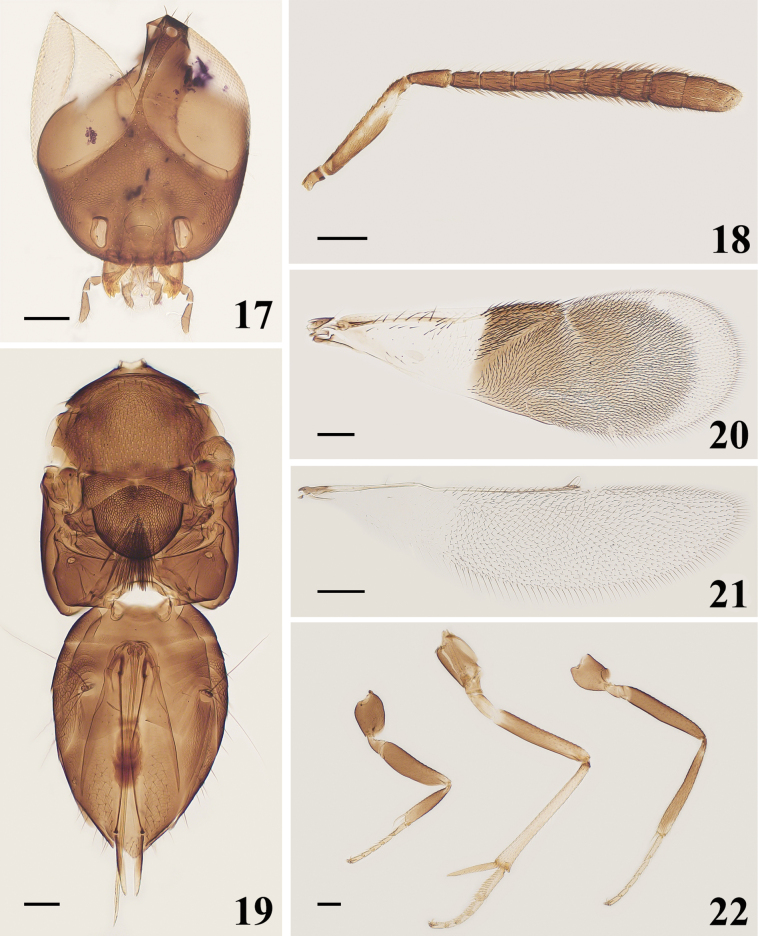
*Cheiloneurusbouceki* ♀ **17** head **18** antenna **19** mesosoma and metasoma **20** fore wing **21** hind wing **22** legs. Scale bars: 100 μm.

**Female**. Length, excluding ovipositor, 1.78–1.89 mm. Body generally dark brown; gena with metallic-bluish green luster, frontovertex metallic-green luster, mandible with three acute teeth (Figs 5–7). Antenna mostly dark brown, an irregular white longitudinal strip in the middle of the scape. Mesosoma dark brown, mesoscutum, axilla, and scutellum with metallic green luster; mesopleuron and propodeum with metallic blue luster. Leg mostly dark brown, basal 1/3 of mid femora, mid tibia, and all tarsi white; frontovertex 0.08–0.12× head width; eye height 1.17–1.33× malar space (Fig. 17); antennal scape 5.05–5.80× as long as wide; pedicel 2–2.19× as long as wide and longer than F1, funicle 6-segmented, F1–F5 longer than width, F6 subquadrate, funicle with linear sensilla on F2–F6; clava 3-segmented, 2.31–2.43× as long as width, shorter than F4–F6 combined (Fig. 18); fore wing 2.78–2.89× as long as wide; linea calva not interrupted and open posteriorly (Fig. 20); ovipositor (Fig. 19) 1.29× as long as mid tibia (Fig. 22), distinctly exserted.

##### Host.

Unknown.

##### Distribution.

China (Guangxi), India.

##### Comments.

This is the first record from China.

#### ﻿ Cheiloneurus chinensis

Taxon classificationAnimaliaHymenopteraEncyrtidae

Shi, Wang, Si & Wang, 1994

EA27220D-D086-5FDA-BA9A-221C5C51872D

Figs 23–25 , 32–38

Cheiloneurus chinensis Shi et al. 1994: 26. Holotype ♀, HAUZ, China, examined plates.

##### Material examined.

China – Henan • 3♀; Gongyi, Luzhuang; 34°37'1"N, 112°52'18"E; 213 m elev.; 15 Jun. 2016; Guo-Hao Zu, Nai-Zhi Li, Jian-Wei Zu leg.; by yellow pan trapping; TJAU-HN-CHE-001 to 003.

##### Diagnosis.

**Female**. Length, excluding ovipositor, 2.4 mm; Antennal (Fig. 33) scape slightly expanded in middle, yellowish brown, ventral margin brown and with an apical one-third white; pedicel brown; F1–F2 brown, F3–F5 white and lower margin brown, F6 dark; clava dark; apical half of msoscutum darker than basal half (Fig. 34); legs (Fig. 38) yellowish brown, except for fore coxa, basal half of fore femora, basal two-thirds of mid femora, hind coxa and basal of tibia yellowish white.

**Figures 23–31.**
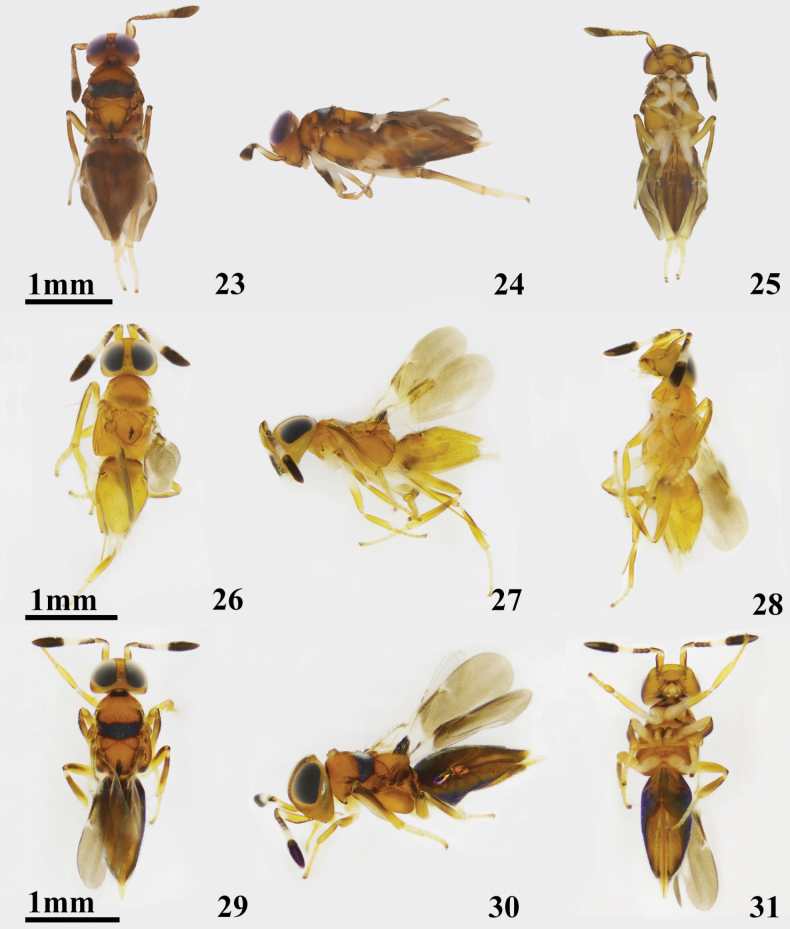
**23–25***Cheiloneuruschinensis* ♀ **23** dorsal habitus **24** lateral habitus **25** ventral habitus **26–31***Cheiloneurusclaviger* ♀ **26** dorsal habitus (Oriental) **27** lateral habitus (Oriental) **28** ventral habitus (Oriental) **29** dorsal habitus (Palaearctic) **30** lateral habitus (Palaearctic) **31** ventral habitus (Palaearctic).

**Figures 32–38.**
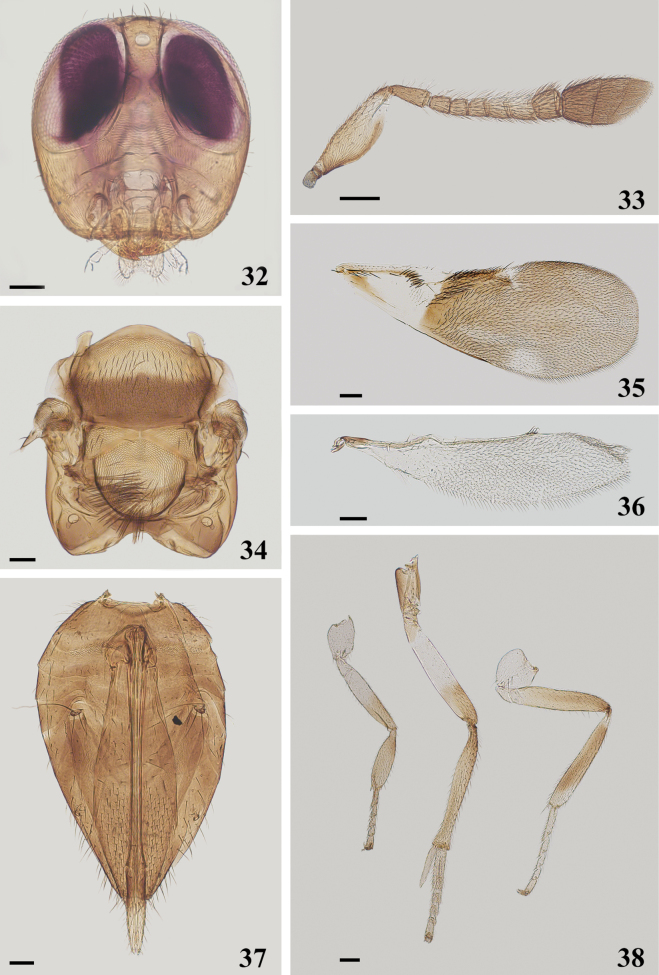
*Cheiloneuruschinensis* ♀ **32** head **33** antenna **34** mesosoma **35** fore wing **36** hind wing **37** metasoma **38** legs. Scale bars: 100 μm.

##### Description.

See Shi et al. (1994).

##### Host.

Coccidae: *Ericeruspela*, *Eulecanium* sp.; Kermesidae: *Kermesquercus* (Xu and Huang 2004).

##### Distribution.

China (Beijing, Liaoning, Henan, Hunan, Hainan, Shandong, Tianjin).

#### ﻿ Cheiloneurus claviger

Taxon classificationAnimaliaHymenopteraEncyrtidae

Thomson, 1876

83A815AB-1C63-5002-AB71-D4A592E446B6

Figs 26–31 , 39–45 , 46–51

Cheiloneurus claviger Thomson 1876: 160. Lectotype ♀, LUZN, Sweden, not examined.
Cheiloneurus japonicus Ashmead 1904: 156. Holotype ♀, USNM, Japan. Synonymized with C.claviger by Japoshvili et al. (2016: 367).
Chiloneurus graeffei Ruschka 1923: 9–10. Holotype ♀, Austria. Synonymized with C.claviger by Claridge (1958: 156–161).
Cheiloneurus claviger Shi 1994: 27–28; Xu and Huang 2003: 104–106, examined plates.

##### Material examined.

China – Liaoning • 1♀; Huludao, Jianchang, Bailong Mountain National Nature Reserve; 40°49'28"N, 119°50'14"E; 716 m elev.; 13 Jul. 2012; Guo-Hao Zu, Ye Chen, Chao Zhang leg.; by yellow pan trapping; TJAU-LN-CHE-001 – Hainan • 1♀; Lingshui Li Autonomous County Diaoluo Mountain; 18°39'35"N, 109°54'57"E; 1499 m elev.; 06 May 2016; Guo-Hao Zu leg.; by yellow pan trapping; TJAU-HN-CHE-001 – Beijing • 4♀; Huairou; 40°18'59"N, 116°37'55"E; 58 m elev.; 20–30 May 2012; Guo-Hao Zu leg.; by Malaise trapping; TJAU-BJ-CHE-001 to 004 – Shandong • 1♀; Qingdao, Cha Mountain National Nature Reserve; 26°52'18"N, 119°51'1"E; 560 m elev.; 13 Jul. 2012; Guo-Hao Zu leg.; by sweep netting; TJAU-SD-CHE-001 – Henan • 3♀; Gongyi, Luzhuang; 34°37'1"N, 112°52'18"E; 213 m elev.; 07 Mar. 2016; Guo-Hao Zu, Nai-Zhi Li, Jian-Wei Zu leg.; by yellow pan trapping; TJAU-HN-CHE-004 – Tianjin • 3♀; Zhangjiawo, Tianjin Academic Agriculture Sciences; 39°6'14"N, 117°3'32"E; 13 m elev.; 29 Oct.–02 Nov. 2021; Guo-Hao Zu, Peng-Hua Bai leg.; by Malaise trapping; TJAU-TJ-CHE-002 to 004.

##### Diagnosis.

**Female**. Length, excluding ovipositor, 1.75–2.42 mm; Head (Fig. 46) brown, antenna dark brown (Fig. 47), except for F3–F5 almost completely white; mandible with three acute teeth; fore wing (Fig. 49) with apical two-thirds dark brown, the basal one-third and the hind wing hyaline; F1–F3 longer than width, F4 subquadrate, F5–F6 2.50× as wide as long.

**Figures 39–45.**
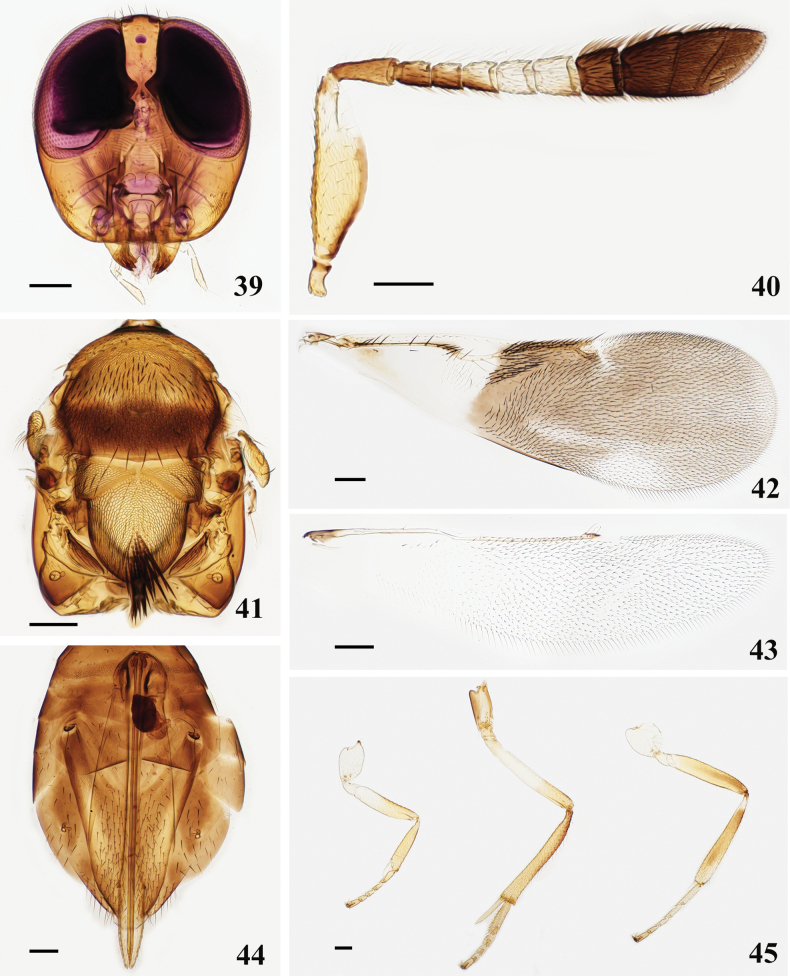
*Cheiloneurusclaviger* (Palaearctic) ♀ **39** head **40** antenna **41** mesosoma **42** fore wing **43** hind wing **44** metasoma **45** legs. Scale bars: 100 μm.

**Figures 46–51.**
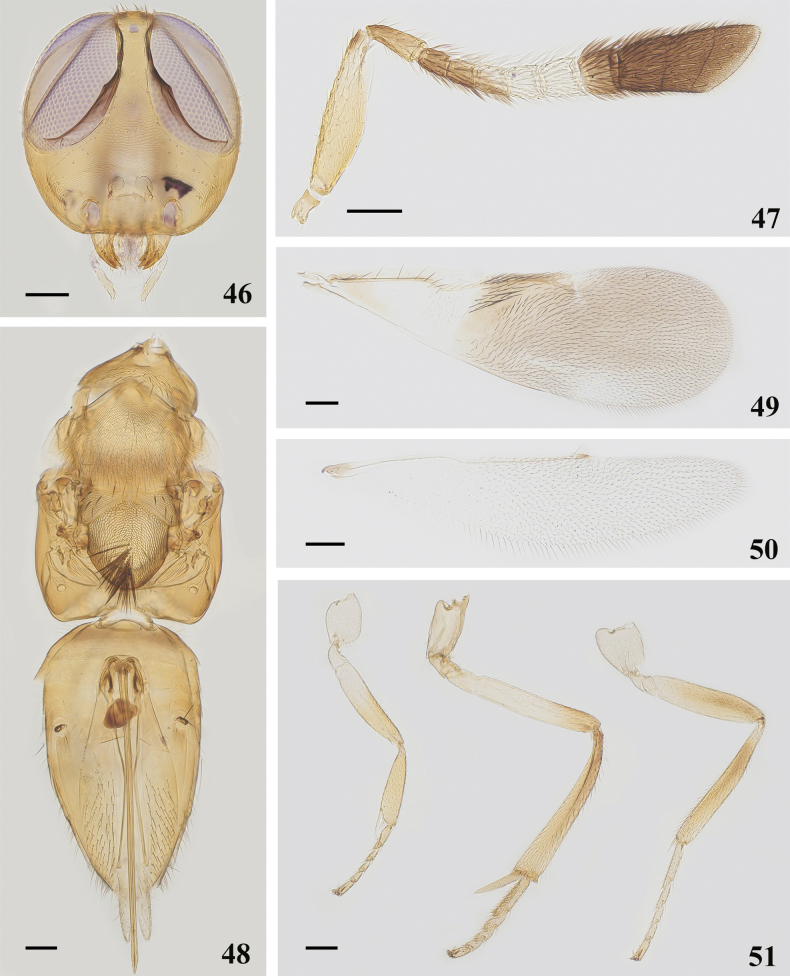
*Cheiloneurusclaviger* (Oriental) ♀ **46** head **47** antenna **48** mesosoma and metasoma **49** fore wing **50** hind wing **51** legs. Scale bars: 100 μm.

##### Description.

See Xu and Huang (2004).

##### Host.

Aphelinidae: *Coccophagusaterrimus*; Encyrtidae: Blastothrixhungarica, *Blastothrixlongipennis*, *Blastothrixscenographica*, *Blastothrixturanica*, *Metaphycusinsidiosus*, *Microterys* sp., *Microteryscuprinus*, *Microterysintermedius*, *Microteryspraedator*, *Microteryssylvius*; Coccidae: *Acanthopulvinariaorientalis*, *Ceroplastesceriferus*, *Ceroplastesjaponicus*, *Chloropulvinariaaurantia*, *Coccushesperidum*, *Didesmococcusunifasciatus*, *Ericeruspela*, *Eulecaniumciliatum*, *Eulecaniumcorni*, *Eulecaniumgiganteum*, *Eulecaniumkunoense*, *Eulecaniumkuwanai*, *Eulecaniumquercifex*, *Eulecaniumrugulosum*, *Eulecaniumtiliae*, *Filippiafolicularis*, *Filippiaviburni*, *Parthenolecaniumcorni*, *Parthenolecaniumpersicae*, *Parthenolecaniumquercifex*, *Parthenolecaniumrufulum*, *Physokermesfasciatus*, *Physokermeshemicryphus*, *Pulvinaria* sp., *Pulvinariaaurantia*, *Pulvinariabetulae*, *Pulvinariaidesiae*, *Pulvinariapopuli*, *Pulvinariavitis*, *Rhizopulvinaria* sp., *Rhodococcusspiraeae*, *Rhodococcusturanicus*, *Saissetiaoleae*, *Sphaerolecaniumprunastri*, *Stotziamaxima*, *Takahashiajaponica*; Eriococcidae: *Eriococcusbrachypodii*, *Greeniscabrachypodii*, *Neoacanthococcustamaricicola*; Kermesidae: *Kermesmiyasakii*, *Kermesvermilio*; Pseudococcidae: *Maconellicoccushirsutus*, *Nesticoccussinensis*, *Nipaecoccusfilamentosus*, *Phenacoccusaceris*, *Phenacoccusmespili*, *Planococcuscitri* (Noyes 2019).

##### Distribution.

China (Liaoning, Hebei, Henan, Shaanxi, Zhejiang, Jiangxi, Hunan, Sichuan, Guangxi), Armenia, Austria, Azerbaijan, Bulgaria, Croatia, Czech Republic, Egypt, France, Georgia, Greece, Hungary, Iran, Israel, Italy, Japan, Kazakhstan, Moldova, Montenegro, Netherlands, Norway, Romania, Russia, Serbia, Slovakia, Spain, Sweden, Tadzhikistan, Turkey, Turkmenistan, Ukraine, England, Uzbekistan.

#### ﻿ Cheiloneurus elegans

Taxon classificationAnimaliaHymenopteraEncyrtidae

(Dalman, 1820)

32736808-C851-5227-B5DC-688C8C5BF4AE

Figs 52–57 , 61–66 , 67–72

Encyrtus elegans Dalman 1820: 151–152. Syntypes, NHRM, Sweden, lost.
Cheiloneurus elegans (Dalman); Westwood 1833: 343.
Cheiloneurus elegantissmus De Santis 1964: 343–345. Holotype ♀, MLP, Argentina, digital image examined, as subspecies of C.elegans (Dalman). Synonymized with C.elegans by Noyes (2023: 372–374).

##### Material examined.

China – Guangxi • 26♀; Qinzhou, Beibu Culf University; 21°53'53"N, 108°36'56"E; 24 m elev.; 09–22 Jun. 2019; Wen-Quan Zhen leg.; by Malaise trapping; TJAU-GX-CHE-027 to 052 – Yunnan • 1♀; Chuxiong Yi Autonomous Prefecture; 25°1'58"N, 101°32'45"E; 1773 m elev.; 15–31 Oct. 2020; Jia-Le Lv leg.; by Malaise trapping; TJAU-YN-CHE-003 – Tianjin • 20♀; Xiqing, Tianjin Agricultural University; 39°5'21"N, 117°5'38"E; 13 m elev.; 14–31 Jul. 2021; Guo-Hao Zu, Ze-Ning Yang leg.; by Malaise trapping; TJAU-TJ-CHE-005 to 024.

##### Description.

**Female**. Length, excluding ovipositor, 1.30–1.89 mm. Head (Figs 61, 67) yellowish brown to brown, mandible with three acute teeth. Antenna (Figs 62, 68) brown to yellow, apex of clava relatively shallow. Mesosoma (Figs 63, 69) yellowish brown, apical half of mesoscutum brown, scutellum yellowish white; fore wing (Figs 64, 70) with apical two-thirds dark brown, basal third and hind wing hyaline (Figs 65, 71); leg yellow (Figs 66, 72), fore coxa, basal half of mid femora, basal third of mid tibial, hind coxa, and basal third of hind tibia white; frontovertex 0.26–0.33× head width; eye height 1.67–2.38× malar space; antennal scape 4.75–5× as long as wide; pedicel 2.09–2.27× as long as wide and longer than F1, funicle 6-segmented, clava 3-segmented, 2.72–2.96× as long as width, longer than F4–F6 combined; fore wing 2.94–4.09× as long as wide; linea calva not interrupted and open posteriorly; ovipositor 1.51–1.63× as long as mid tibia, slightly exserted.

**Figures 52–60.**
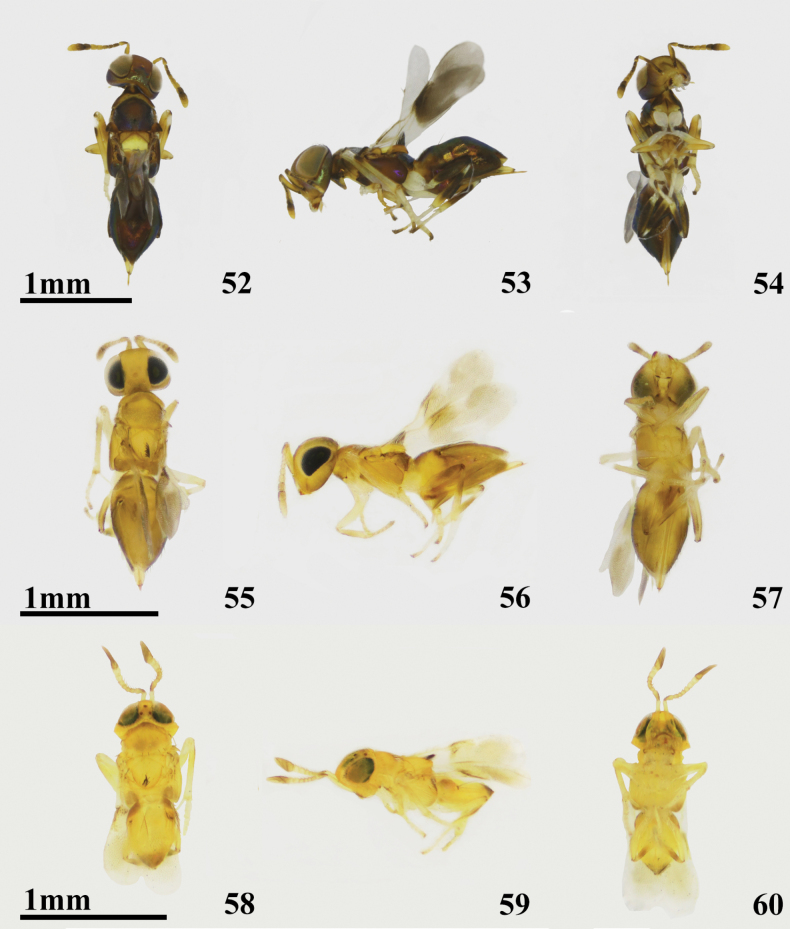
*Cheiloneuruselegans* ♀ **52** dorsal habitus (Palaearctic) **53** lateral habitus (Palaearctic) **54** ventral habitus (Palaearctic) **55** dorsal habitus (Oriental) **56** lateral habitus (Oriental) **57** ventral habitus (Oriental) **58–60***Cheiloneurusgonatopodis* ♀ **58** dorsal habitus **59** lateral habitus **60** ventral habitus.

**Figures 61–66.**
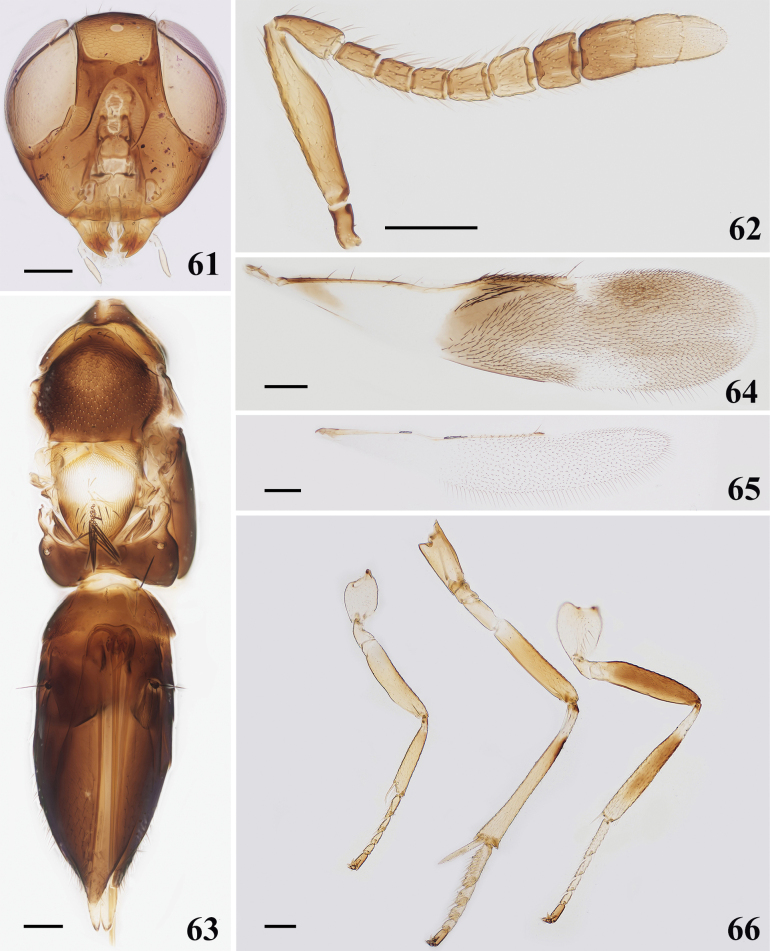
*Cheiloneuruselegans* (Palaearctic) ♀ **61** head **62** antenna **63** mesosoma and metasoma **64** fore wing **65** hind wing **66** legs. Scale bars: 100 μm.

**Figures 67–72.**
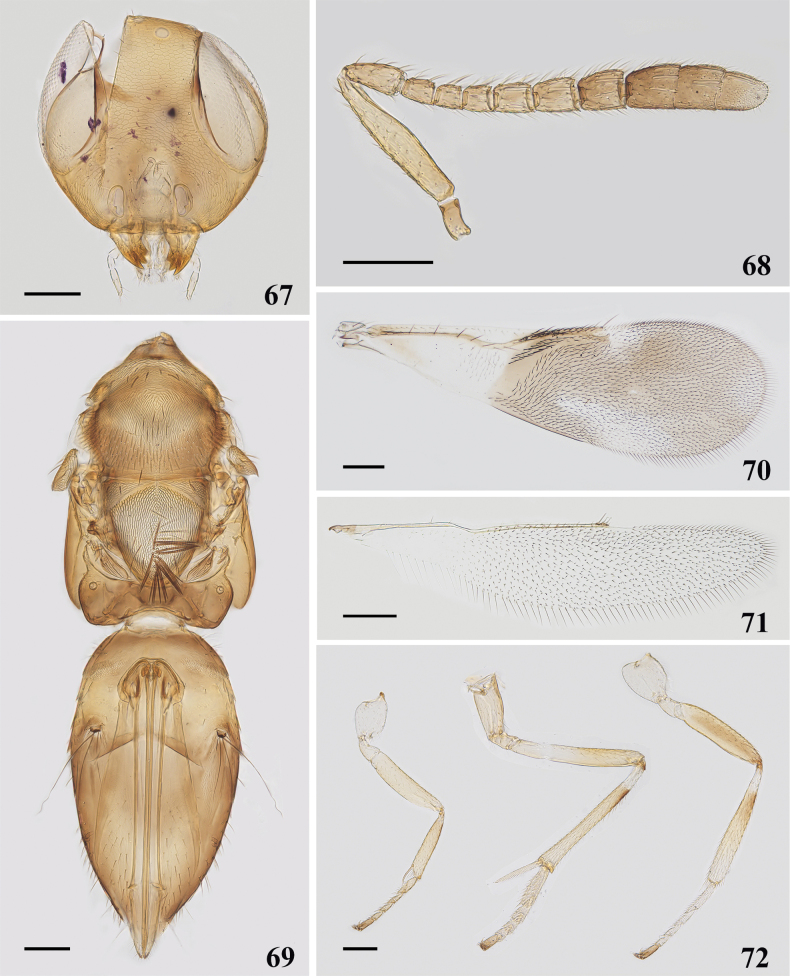
*Cheiloneuruselegans* (Oriental) ♀ **67** head **68** antenna **69** mesosoma and metasoma **70** fore wing **71** hind wing **72** legs. Scale bars: 100 μm.

##### Variation.

There is significant variation in body color, size of F5–F6, and fore wings between specimens collected in the Palaearctic and in the Oriental realms. The specimens from the Palaearctic have a darker body, F5–F6 are longer than wide (Fig. 33), and the fore wing is 4.09× as long as wide, while Oriental specimens have a relatively lighter body color, F5–F6 are wider than long (Fig. 34), and the fore wing is 2.94× as long as wide.

##### Host.

Encyrtidae: *Epidinocarsislopezi*; Platygastridae: *Platygasterzosine*; Cecidomyiidae: *Mayetioladestructor*, *Phytophagadestructor*; Aclerdidae: *Aclerdasubterranean*; Coccidae: *Anapulvinariapistaciae*, *Eulecaniumfranconicum*, *Physokermespiceae*, *Pulvinariavitis*, Kermesidae: *Kermes* sp.; Pseudococcidae: *Antoninapurpurea*, *Phenacoccushordei*, *Phenacoccusmanihoti*, *Trionymusaberrans* (Noyes 2019).

##### Distribution.

China (Tianjin, Guangxi, Yunnan), America, Argentina, Armenia, Austria, Azerbaijan, Bulgaria, Canada, Croatia, Denmark, England, Finland, France, Georgia, Germany, Hungary, India, Israel, Italy, Kazakhstan, Lithuania, North Macedonia, Mexico, Moldova, Mongolia, Netherlands, Nigeria, Romania, Russia, Saudi Arabia, Serbia, Spain, Sweden, Switzerland, Tadzhikistan, Transcaucasus, Turkey, Turkmenistan, Ukraine, Uzbekistan.

#### ﻿ Cheiloneurus exitiosus

Taxon classificationAnimaliaHymenopteraEncyrtidae

(Perkins, 1906)

CF67F1B3-A182-5E2C-A03A-0FA04FF40311

Echthrogonatopus exitiosus Perkins, in Perkins et al. 1906: 256. Holotype ♀, BPBM, Australia, not examined.
Metapterencyrtus nigricornis Hayat 1980: 644. Holotype ♀, ZDANU, India. Synonymized with exitiosus by Guerrieri and Viggiani (2005: 305–317).
Echthrogonatopus nigricornis (Hayat); Hayat 1981: 20; Xu and He 2003: 527, examined plates.
Cheiloneurus exitiosus (Perkins); Guerrieri and Viggiani 2005: 305.

##### Diagnosis.

Body dark brown, antennae dark, mesoscutum and axilla with metallic-green luster, scutellum without a tuft of bristles at apex, leg yellowish white, mid coxa dark basally.

##### Description.

See Xu and He (2003).

##### Host.

Bethylidae: *Goniozus* sp.; Dryinidae: *Dryinidaeunspecified* sp., *Gonatopus* sp., *Haplogonatopus* sp., *Haplogonatopusvitiensis*, *Pseudogonatopusflavifemur*, *Pseudogonatopushospes*, *Pseudogonatopusperkinsi*; Delphacidae: *Megamelusproserpina*, *Nilaparvatalugens*, *Sogata* sp., *Sogatafurcifera*, *Sogatellafurcifera*, Pyralidae: *Cnaphalocrocismedinalis*, *Marasmiaexigua* (Guerrieri and Viggiani 2005).

##### Distribution.

China (Fujian, Zhejiang, Jiangxi, Guangxi), American, Australia, Fiji, Guam, India, Malaysia, Philippines.

#### ﻿ Cheiloneurus gonatopodis

Taxon classificationAnimaliaHymenopteraEncyrtidae

Perkins, 1906

210B30C8-12C9-55CA-B6D9-9E9282D4C345

Figs 58–60 , 73–77

Cheiloneurus gonatopodis Perkins, in Perkins et al. 1906: 261. Lectotype ♀ designated by Noyes 1988: 63: Australia, Queensland, Childers (BPBM).
Cheiloneurus gonatopodis Perkins; Anis and Hayat 2002: 152; Guerrieri and Viggiani 2005: 310.

##### Material examined.

China – Hainan • 1♀; Lingshui Li Autonomous County, Diaoluo Mountain; 18°39'35"N, 109°54'57"E; 1499 m elev.; 15 May 2016; Guo-Hao Zu leg.; by yellow pan trapping; TJAU-HN-CHE-005.

##### Description.

**Female**. Length, excluding ovipositor, 1.16 mm. Head yellow, frontovertex brown. Antenna yellowish brown, scape yellow. Mesosoma yellowish brown, leg yellowish white, basal half of hind tibial white. Metasoma mostly yellow, but basally and apically brown, frontovertex 0.21× head width; eye height 1.62× malar space; antennal (Fig. 74) scape 6.38× as long as wide; pedicel 2.23× as long as wide and longer than F1–F2 combined, funicle 6-segmented, clava 3-segmented, 2.6× as long as width, shorter than F2–F6 combined; fore wing (Fig. 76) 2.96× as long as wide; linea calva not interrupted and open posteriorly; mid tibial spur (Fig. 77) 0.31× as long as mid tibia and shorter than basitarsus, ovipositor (Fig. 75) as long as mid tibia, not exserted.

**Figures 73–77.**
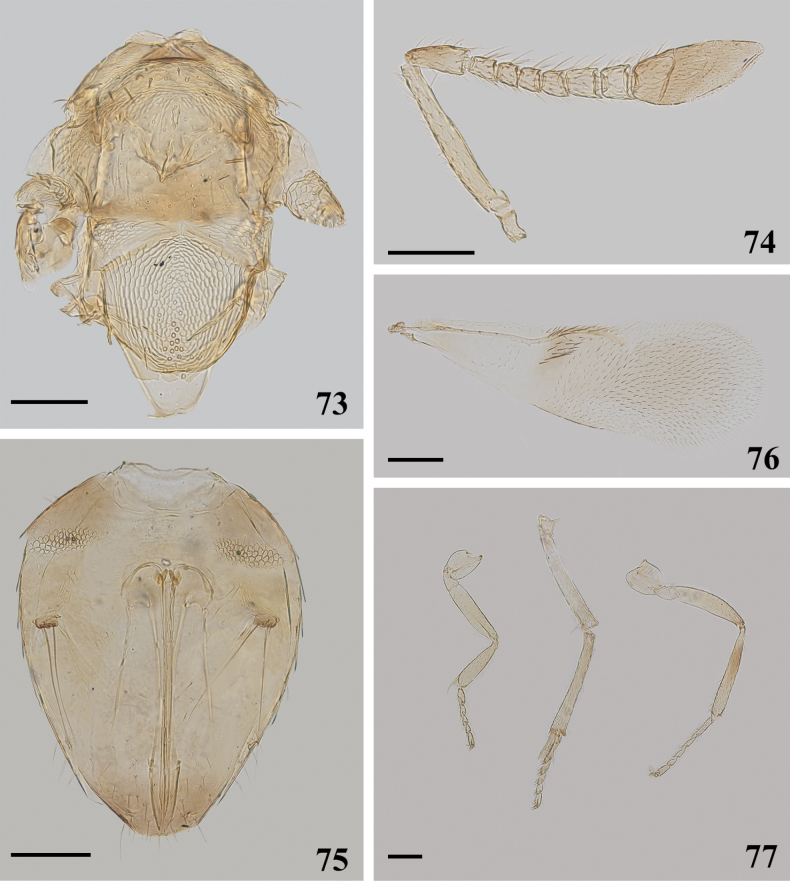
*Cheiloneurusgonatopodis* ♀ **73** mesosoma **74** antenna **75** metasoma **76** fore wing **77** legs. Scale bars: 100 μm.

##### Host.

Dryinidae: *Echthrodelphax* sp., *Pseudogonatopoidesmauritianus*, *Pseudogonatopus* sp., *Pseudogonatopusmauritianus*, *Richardsidryinus* sp.; Delphacidae: *Dicranotropismuiri*, *Nilaparvatamyersi*, *Perkinsiellasaccharicida* (Guerrieri and Viggiani 2005).

##### Distribution.

China (Hainan), Afrotropical, Australia, India, Madagascar, Mauritius, New Zealand.

##### Comments.

This is the first record from China.

#### ﻿ Cheiloneurus guangxiensis

Taxon classificationAnimaliaHymenopteraEncyrtidae

Zu sp. nov.

B2B2F582-4F38-566A-8EAE-3B55E023D902

https://zoobank.org/BDEFCF90-E941-4AC9-B6A6-EA4C172B2EA4

Figs 78–83

##### Type material.

***Holotype***. ♀, [on slide]; China – Guangxi, Qinzhou, Beibu Gulf University; 21°53'53"N, 108°36'56"E; 24 m elev.; 14–24 Dec. 2019; Wen-Quan Zhen leg.; by Malaise trapping; TJAU-GX-CHE-053. ***Paratype***. 1♀; same date as holotype; TJAU-GX-CHE-054.

##### Description.

**Female**. Holotype. Length, 1.51 mm (excluding ovipositor). Head with yellow to yellowish brown and metallic-green luster, basal half of antennal scape brown, apical half white, and dorsal margin brown; pedicel brown; F1 brown with dorsal margin white, F2–F4 lower margin brown and dorsal margin white, F5 lower margin slightly brown and dorsal margin white, F6 white and small part of lower margin brown; clava dark brown. Mesosoma yellow; leg white, nearly transparent, basal third of mid tibia and apical fifth of hind femora brown. Metasoma dark brown, with metallic-blue luster, but slightly yellowish brown in middle and lower part.

**Figures 78–83.**
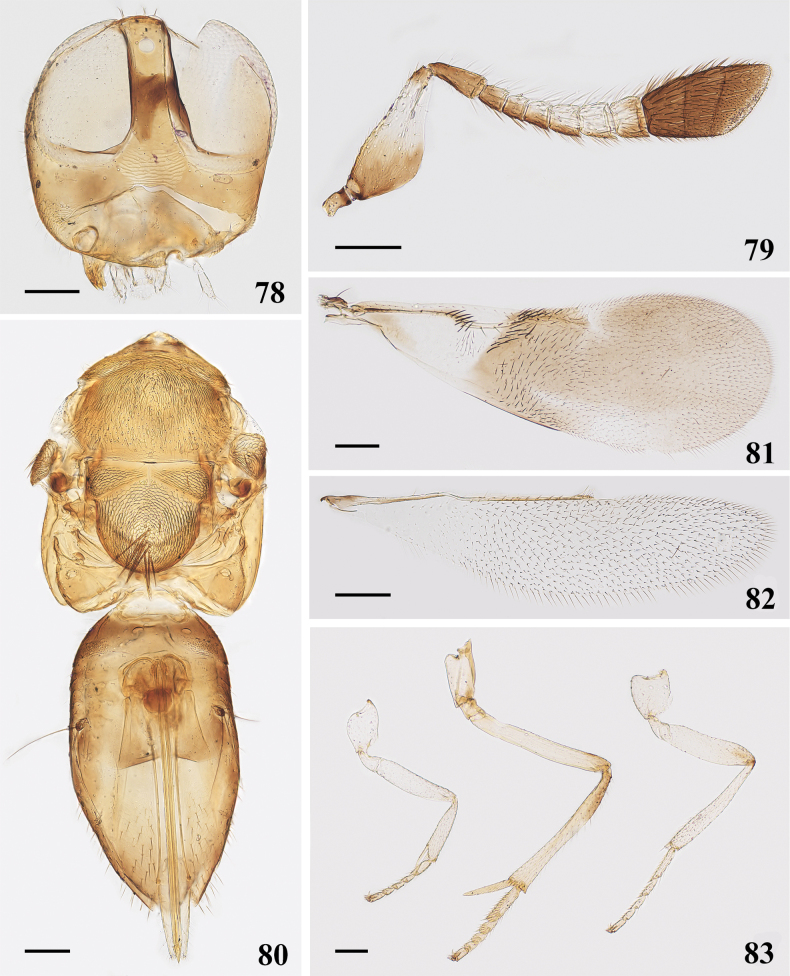
*Cheiloneurusguangxiensis* sp. nov. ♀ holotype **78** head **79** antenna **80** mesosoma and metasoma **81** fore wing **82** hind wing **83** legs. Scale bars: 100 μm.

Head (Fig. 78) in frontal view, length equal to width, frontovertex 0.14× head width; ocelli forming an angle of 40°, OCL about 1.67× diameter of posterior ocellus, OOL about 0.10× the diameter of posterior ocellus; antennal torulus with its dorsal margin well above lower margin of eye; eye length about 1.77× as long as malar space. Antennal (Fig. 79) scape flattened and expanded, about 2.37× as long as width, pedicel about 2.26× as long as wide, funicle 6-segmented, F1 longer than width, F2–F3 subquadrate, F4–F6 wider than long, clava 3-segmented, 1.98× as long as wide, longer than F3–F6 combined, funicle with linear sensillae on F3–F6. Mandible with three acute teeth. Measurements (μm): HH, 460; HW, 460; FV, 63; OD, 30; POL, 23; OOL,3; OCL, 50; AOL, 43; EL, 270; MS, 153; length (and width): radicle, 45; scape, 225 (95); pedicel, 88 (39); F1, 38 (35); F2, 35 (35); F3, 38 (38); F4, 45 (48); F5, 48 (55); F6, 46 (63); clava, 206 (103).

Mesosoma (Fig. 80). Mesoscutum densely setose; mesoscutum and scutellum with finely reticulate sculpture, mesoscutum 0.60× as long as wide, scutellum 0.90× as long as wide and with a tuft of bristles at apex. Fore wing (Fig. 81) 2.70× as long as wide, apical half of submarginal vein strongly curved, marginal vein longer than width, and 4.91× as long as postmarginal vein, linea calva not interrupted and open posteriorly, hind wing (Fig. 82) 4.32× as long as width. Mid tibial spur (Fig. 83) 0.35× as long as mid tibia and longer than basitarsus. Measurements (μm): FWL, 960; FWW, 355; submarginal vein, 350; MV, 113; PMV, 23; SV, 45; HWL, 800; HWW,185; MT, 450; mid tibial spur, 163; mid basitarsus, 140.

Metasoma (Fig. 80) slightly longer than mesosoma, ovipositor 1.51× as long as mid tibia, distinctly exserted. Measurements (μm): OL, 680. [MT, 450].

**Male.** Unknown.

##### Host.

Unknown.

##### Etymology.

The specific name refers to the province where the type locality is located.

##### Diagnosis.

The new species is similar to *C.chinensis* Shi, Wang, Si & Wang, 1994 but differs from *C.chinensis* as follows: frontovertex 0.14× head width (0.20× in *chinensis*); scape flattened and expanded, about 2.37× as long as wide (scape 3× as long as wide in *chinensis*); F6 white and small part of the lower margin brown (F6 black in *chinensis*); clava longer than F3–F6 combined (nearly equal length F3–F6 combined in *chinensis*); legs off white nearly transparent, mid tibia basal one-third and hind femora apical one-fifth brown (legs brownish yellow except fore tibia; apical third of mid feroma, outer margin of hind feroma and tibia brown in *chinensis*).

#### ﻿ Cheiloneurus hadrodorys

Taxon classificationAnimaliaHymenopteraEncyrtidae

Anis & Hayat, 2002

7D86FEEC-796F-50B7-93D4-3672777F14F3

Figs 84–86 , 93–98

Cheiloneurus hadrodorys Anis and Hayat 2002: 138, 173–175. Holotype ♀, BMNH, Nepal.

##### Material examined.

China – Guangxi • 6♀; Qinzhou, Beibu Culf University; 21°53'53"N, 108°36'56"E; 24 m elev.; 11–18 May 2019, 04–13 Jan. 2020; Wen-Quan Zhen leg.; by Malaise trapping; TJAU-GX-CHE-055 to 060 – Yunnan • 1♀; Chuxiong Yi Autonomous Prefecture; 25°1'58"N, 101°32'45"E; 1773 m elev.; 01–15 Jun. 2022; Jia-Le Lv; by Malaise trapping; TJAU-YN-CHE-004.

##### Description.

**Female**. Length, excluding ovipositor, 1.67–1.89 mm. Body generally brown; gena with metallic-green luster, frontovertex dark brown, mandible with three acute teeth. Antennal radicle brown, scape yellow, and inner margin brown, pedicel and F1–F3 with brown, F4 yellowish white, F5 brown, F6 and clava with dark brown. Pronotum mostly brown, but left and right margin with yellow; mesoscutum dark brown; axilla, scutellum and mesopleuron yellow, propodeum yellow, left and right margin dark brown with metallic-green luster. Leg yellowish white, except apical half of hind femora dark brown. Metasoma mostly brown to dark brown and basal quarter yellow; frontovertex (Fig. 93) 0.24–0.27× head width; eye height 2.13–2.5× malar space; antennal (Fig. 94) scape 5–5.48× as long as wide; pedicel 2.07× as long as wide and longer than F1, funicle 6-segmented, with F1–F6 widening gradually, clava 3-segmented, 2.25–2.25× as long as wide, longer than F4–F6 combined; fore wing (Fig. 96) 3.47–3.71× as long as wide; linea calva closed posteriorly by several lines of setae; mid tibial spur (Fig. 98) 0.36–0.38× as long as mid tibia and longer than basitarsus, ovipositor (Fig. 95) 1.86–2.07× as long as mid tibia, strongly exserted.

**Figures 84–92.**
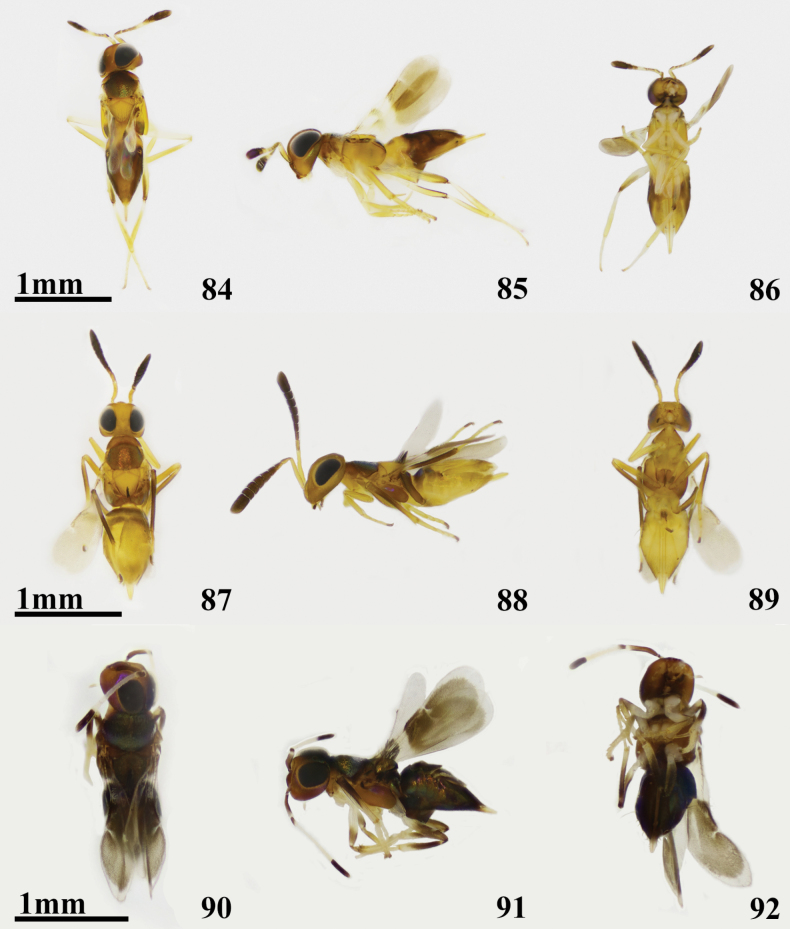
**84–86***Cheiloneurushadrodorys* ♀ **84** dorsal habitus **85** lateral habitus **86** ventral habitus **87–89***Cheiloneurusnankingensis* ♀ **87** dorsal habitus **88** lateral habitus **89** ventral habitus **90–92***Cheiloneurusquercus* ♀ **90** dorsal habitus **91** lateral habitus **92** ventral habitus.

**Figures 93–98.**
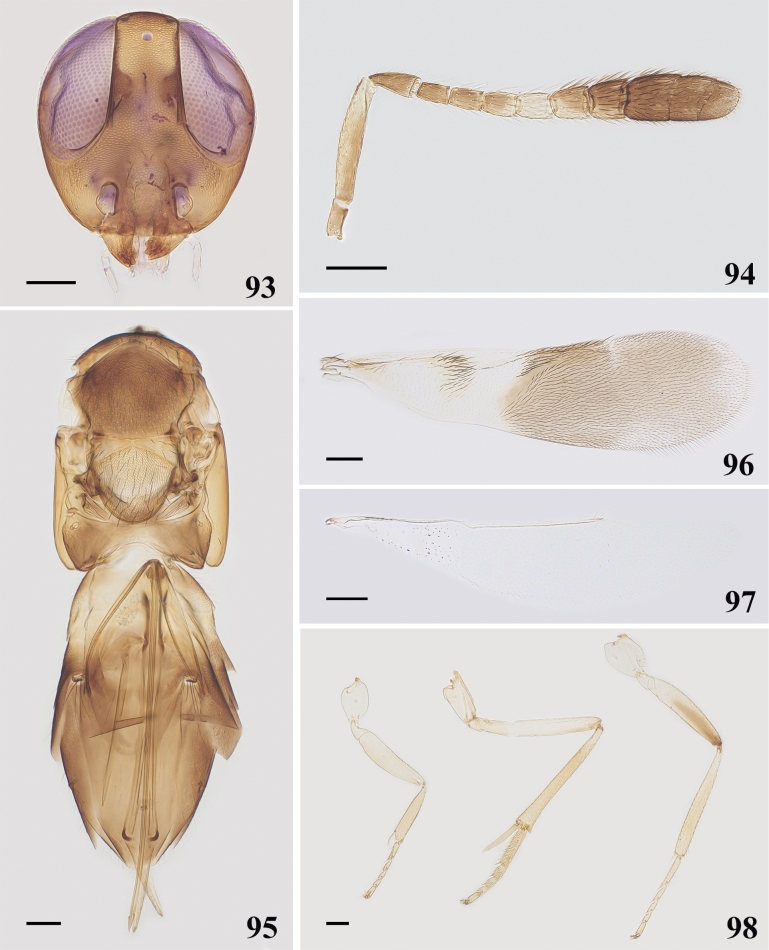
*Cheiloneurushadrodorys* ♀ **93** head **94** antenna **95** mesosoma and metasoma **96** fore wing **97** hind wing **98** legs. Scale bars: 100 μm.

##### Host.

Pseudococcidae: *Saccharicoccussacchari* (Anis and Hayat 2002).

##### Distribution.

China (Yunnan, Guangxi), India, Nepal, Pakistan, Sri Lanka.

##### Comments.

This is the first record from China.

#### ﻿ Cheiloneurus lateocaudatus

Taxon classificationAnimaliaHymenopteraEncyrtidae

(Xu & He, 2003)

759EAD9E-D6FE-5F0B-9E0D-D65656E60027

Echthrogonatopus lateocaudatus Xu and He 2003: 527. Holotype ♀. ZAUC, China, examined plates.

##### Diagnosis.

Body dark, head with dark metallic-blue luster; antennal dark brown dark; scutellum without a tuft of bristles at apex; fore wing hyaline; leg yellowish white but base of mid coxa dark; F1 1.1× as long as wide; F2 and F5 subquadrate; F3–F4 and F6 wider than long; clava slightly shorter than F1–F6 combined.

##### Description.

See Xu and He (2003).

##### Host.

Dryinidae: *Haplogonatopusapicalis*, *Haplogonatopusoratorius* (Xu and He 2003).

##### Distribution.

China (Anhui, Fujian, Guangdong, Guangxi, Guizhou, Hubei, Hunan, Jiangsu, Jiangxi, Shanghai, Sichun, Yunnan, Zhejiang).

#### ﻿ Cheiloneurus nankingensis

Taxon classificationAnimaliaHymenopteraEncyrtidae

Li & Xu, 2020

7D3DE27E-4E1B-59DA-8AA1-9F78EEF1F470

Figs 87–89 , 99–104 , 105–106

Cheiloneurus nankingensis Li and Xu 2020: 23. Holotype ♀, ZAFU, China; digital image examined.

##### Material examined.

China – Guangxi • 6♀; Qinzhou, Beibu Culf University; 21°53'53"N, 108°36'56"E; 24 m elev.; 02–17 Nov. 2019, 01–29 Dec. 2019; Wen-Quan Zhen leg.; by Malaise trapping; TJAU-GX-CHE-061 to 066 – Jiangxi • 6♀, 2♂; Jiujiang, De’an; 29°16'6"N, 115°22'38"E; 64 m elev.; 17–19 Aug. 2020, 06–07 Sep. 2020; Yan-Yan Qiao leg.; ex. *Aenasiusarizonensis* on *Phenacoccussolenopsis*; TJAU-JX-CHE-001 to 008 – Jiangsu • 2♂; Nanjing, Nanjing Agricultural University; 32°01'10"N, 118°51'21"E; 18 m elev.; 01–31 Oct. 2019; Zhuo-Miao Li leg.; ex. *Aenasiusarizonensis* on *Phenacoccussolenopsis*; TJAU-JS-CHE-001 to 002.

##### Diagnosis.

**Figures 99–104.**
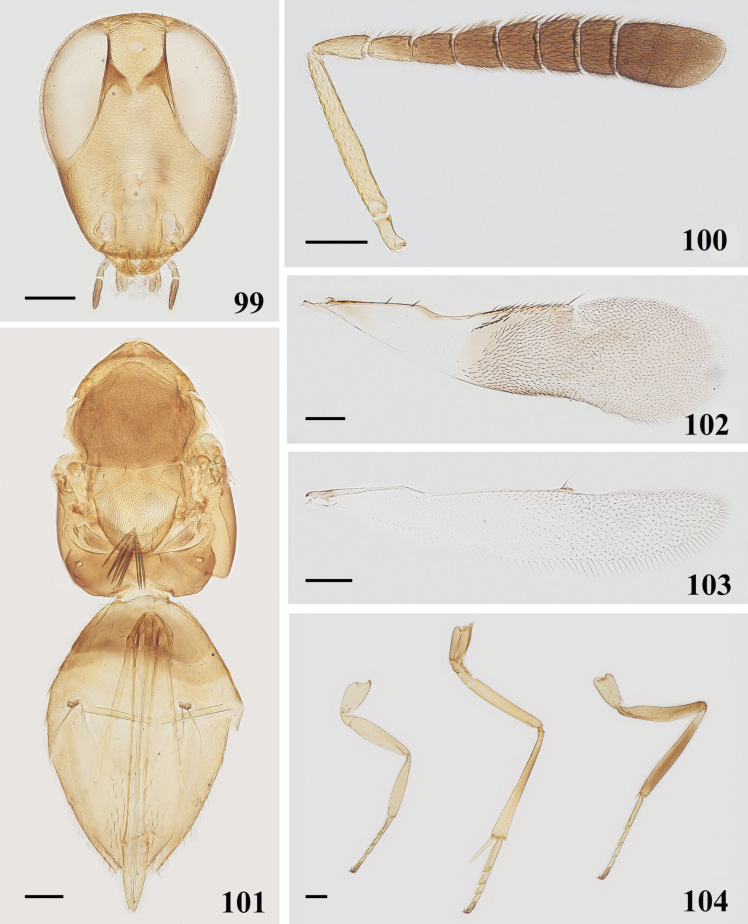
*Cheiloneurusnankingensis* ♀ **99** head **100** antenna **101** mesosoma and metasoma **102** fore wing **103** hind wing **104** legs. Scale bars: 100 μm.

**Female.** Length, excluding ovipositor, 1.54–1.75 mm; head (Fig. 99) dark brown, with purple sheen; antennal (Fig. 100) scape and pedicel yellowish brown; funicle and clava dark; fore and mid legs yellowish brown, except middle part of mid tibia brown; hind femur and tibia brown, except tibial base white.

##### Description.

See Li et al. (2020).

##### Host.

Encyrtidae: *Aenasiusarizonensis*; Pseudococcidae: *Phenacoccussolenopsis* (Li et al. 2020).

##### Distribution.

China (Jiangsu, Jiangxi, Guangxi).

##### Comments.

This species is very similar to *C.compressicommis* (Ashmead, 1894). After comparing with the original description and the text description and figures by Noyes (2023), it was found that *C.nankingensis* is only slightly different from *C.compressicommis* in the color of the male forewing. For this reason, we examined the paratype specimens from Nanjing and the specimens collected from Jiangxi, and reconfirmed the above differences. Therefore, we maintain the same view as Noyes, and the species is still designated as *C.nankingensis* here.

#### ﻿ Cheiloneurus quercus

Taxon classificationAnimaliaHymenopteraEncyrtidae

Mayr, 1876

941D7AFE-2DED-5283-BAC6-85441F80F6C0

Figs 90–92 , 107–113

Cheiloneurus quercus Mayr 1876: 744, 746, Austria, not examined.
Cheiloneurus tenuicornis Ishii 1928: 147–148. Lectotype ♀, NIES, Japan. Synonymized with C.quercus by Trjapitzin (1989: 305).
Cheiloneurus quercus Japoshvili et al. 2016: 368.

##### Material examined.

China – Henan •1♀; Gongyi, Luzhuang; 34°37'1"N, 112°52'18"E; 213 m elev.; 07 May 2016; Guo-Hao Zu, Nai-Zhi Li, Jian-Wei Zu leg.; by yellow pan trapping; TJAU-HN-CHE-006 – Tianjin • 1♀; Jixian, Baxian Mountain National Nature Reserve; 40°11'58"N, 117°33'52"E; 1052 m elev.; 01 Oct. 2023; Ke-Long Jiao leg.; by sweep netting; TJAU-TJ-CHE-025.

##### Diagnosis.

**Female**. Length, excluding ovipositor, 1.71–2.0 mm; antennal scape brown, apex white, dorsal margin of pedicel brown, ventral margin of pedicel and all funiculars white, clava dark; mandible with one tooth and a broadly truncate upper tooth; legs pale; all femora apically light brown; basal half of all tibia light brown; metasoma forming a long triangle, slightly shorter and narrower than mesosoma; ovipositor slightly exserted.

**Figures 105, 106.**
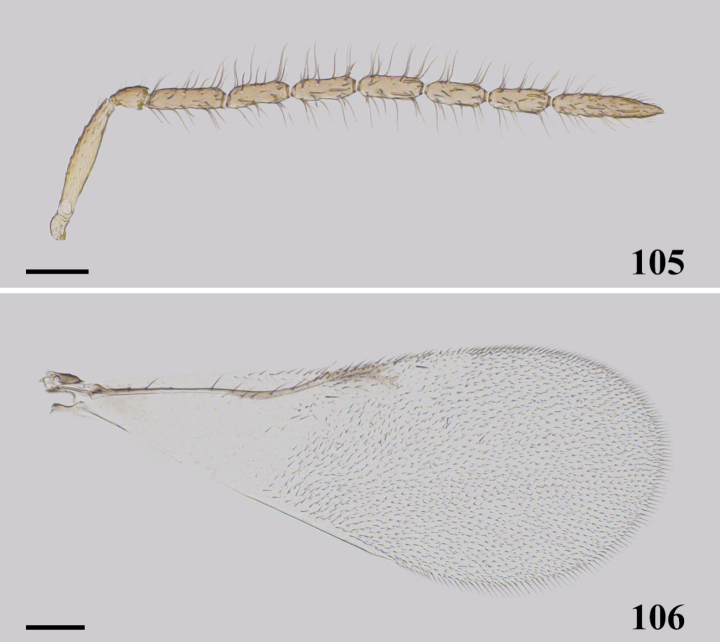
*Cheiloneurusnankingensis* ♂ **105** antenna **106** fore wing. Scale bars: 100 μm.

**Figures 107–113.**
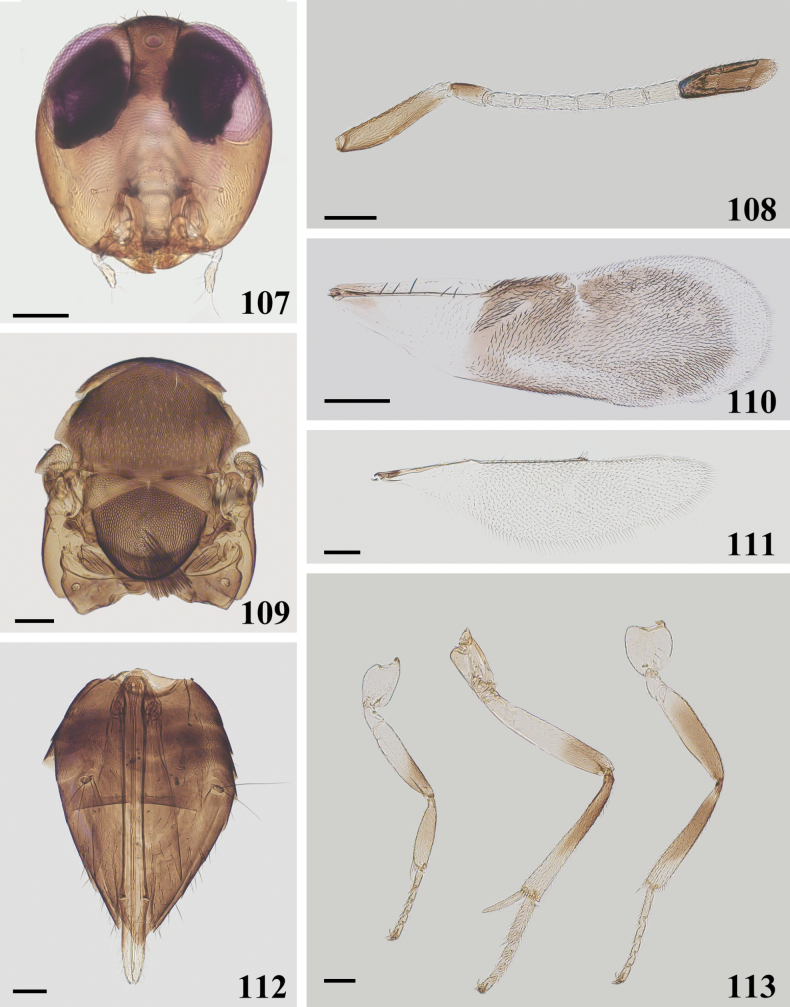
*Cheiloneurusquercus* ♀ **107** head **108** antenna **109** mesosoma **110** fore wing **111** hind wing **112** metasoma **113** legs. Scale bars: 100 μm.

##### Description.

See Ma (2004).

##### Host.

Coccidae: *Eulecanium* sp., *Pulvinariavitis*, Kermesidae: *Kermesmiyasakii*, *Kermesnakagawae*, *Kermesquercus*, *Kermococcus* sp., *Kermococcusmiyasakii*, *Kermococcusnakagawae*, Pseudococcidae: *Coccuraussuriensis*, *Phenacoccuspolyphagus* (Noyes 2019).

##### Distribution.

China (Liaoning, Tianjin, Henan, Shaanxi, Shandong), Austria, Czech Republic, Hungary, Italy, Japan, Russia, Turkey.

#### ﻿ Cheiloneurus sinensis

Taxon classificationAnimaliaHymenopteraEncyrtidae

Özdikmen, 2011

E4D4B77E-554C-5762-A26D-97EBA564B3EC

Cheiloneurus phenacocci Shi, in Shi et al. 1994: 25. Holotype ♀, HAUZ, China; not examined.
Cheiloneurus sinensis Ozdikmen 2011: 801.

##### Diagnosis.

**Female**. Antennal scape light brown, basal half of pedicel dark and apical half white; funicle white; clava white; mesosoma brown; axilla yellowish brown, mid tibial spur as long as basitarsus.

##### Description.

See Shi et al. (1994).

##### Host.

Pseudococcidae: *Phenacoccusflaxinus* (Shi et al. 1994).

##### Distribution.

China (Henan, Shaanxi).

## Supplementary Material

XML Treatment for Cheiloneurus axillaris

XML Treatment for Cheiloneurus boldyrevi

XML Treatment for Cheiloneurus bouceki

XML Treatment for Cheiloneurus chinensis

XML Treatment for Cheiloneurus claviger

XML Treatment for Cheiloneurus elegans

XML Treatment for Cheiloneurus exitiosus

XML Treatment for Cheiloneurus gonatopodis

XML Treatment for Cheiloneurus guangxiensis

XML Treatment for Cheiloneurus hadrodorys

XML Treatment for Cheiloneurus lateocaudatus

XML Treatment for Cheiloneurus nankingensis

XML Treatment for Cheiloneurus quercus

XML Treatment for Cheiloneurus sinensis

## ﻿Additional information

### Author ORCIDs

Haiyang Wang https://orcid.org/0009-0007-5665-2111

Wenyu Cui https://orcid.org/0009-0003-2930-9028

Guohao Zu https://orcid.org/0000-0002-9892-2171

### Data availability

All of the data that support the findings of this study are available in the main text.
